# IgA in human health and diseases: Potential regulator of commensal microbiota

**DOI:** 10.3389/fimmu.2022.1024330

**Published:** 2022-11-10

**Authors:** Tadashi Takeuchi, Hiroshi Ohno

**Affiliations:** ^1^ Laboratory for Intestinal Ecosystem, RIKEN Center for Integrative Medical Sciences, Yokohama, Japan; ^2^ Graduate School of Medical Life Science, Yokohama City University, Yokohama, Japan

**Keywords:** immunoglobulin A, gut microbiota, microbial metabolite, human immunology, adaptive immunity

## Abstract

Gut microbiota has extensive and tremendous impacts on human physiology and pathology. The regulation of microbiota is therefore a cardinal problem for the mutualistic relationship, as both microbial overgrowth and excessive immune reactions toward them could potentially be detrimental to host homeostasis. Growing evidence suggests that IgA, the most dominant secretory immunoglobulin in the intestine, regulates the colonization of commensal microbiota, and consequently, the microbiota-mediated intestinal and extra-intestinal diseases. In this review, we discuss the interactions between IgA and gut microbiota particularly relevant to human pathophysiology. We review current knowledge about how IgA regulates gut microbiota in humans and about the molecular mechanisms behind this interaction. We further discuss the potential role of IgA in regulating human diseases by extrapolating experimental findings, suggesting that IgA can be a future therapeutic strategy that functionally modulates gut microbiota.

## Introduction

IgA is the most dominant immunoglobulin class in humans. IgA-producing cells are mainly distributed in the mucosa lining tissues such as the intestine and oral and nasal cavities. IgA produced by these cells are usually dimerized within the cells through disulfide bonds of its C-terminus with the joining chain (J chain). Dimeric IgA is bound with polymeric Ig receptor (pIgR) on the basolateral surface of intestinal epithelial cells and endocytosed. During transcytosis through intestinal epithelial cells, the extracellular domain of pIgR is cleaved by endopeptidase in the transport vesicle, and this domain, now called secretary component (SC), is secreted with bound IgA to the intestinal lumen. IgA complexed with SC is designated as secretory IgA (SIgA). SIgA is protected from bacterial proteases thanks to the presence of SC, and thus has a longer half-life than IgA (1).

The targets of IgA are “foreign bodies” for us, such as microbes, dietary antigens, etc. In the past decades, IgA has been studied as a cardinal component of mucosal defense against pathogens such as genera *Salmonella* and *Vibrio* based on the assumption that the control of pathogens is the primary role of IgA (2–6). More recently, many studies have also appreciated the role of IgA in regulating gut commensal microbiome (7, 8). Surprisingly, however, the lack of IgA in humans rarely causes severe complications such as infectious diseases (9, 10), in part because of compensation by other immunoglobulin classes such as IgM (11, 12). Nevertheless, growing evidence also links IgA deficiency and altered microbiome in humans (11, 13). Considering the tremendous impact of the human microbiome on health and diseases, ranging from local intestinal diseases to systemic disorders relating to the central nervous system, systemic metabolism, and autoimmunity, it is tempting to presume that regulation of the microbiome by IgA may modulate the susceptibility of these human diseases other than infections. In addition, understanding the biology of IgA may lead to a novel opportunity to artificially modulate the susceptibility for the diseases through microbiome regulation. In this review, we summarize the current knowledge regarding the relationship between IgA and gut microbiome and discuss how IgA controls the microbiome and, as a consequence, our health.

## The role of IgA in humans: What is known and unknown?

IgA has long been studied in the context of infectious diseases and their pathogens, such as genera *Salmonella* and *Vibrio*, their toxins like Cholera toxin. For example, IgA can bind to *Salmonella* and suppress their motility (14) and invasion (5). IgA also facilitates agglutination, enchaining, and therefore, clearance of *Salmonella* (4). In addition, IgA can neutralize Cholera toxin and mitigate its pathogenicity (6). IgA not only directly acts on these pathogens to limit their pathogenicity but also facilitates immune reactions against these pathogens through retro transcytosis (15). In this transport mechanism, IgA binds to antigens such as bacteria and then bind to surface receptors of microfold cells (M cells) such as Dectin-1 on the Peyer’s patches (15), although a later study shows that Dectin-1 is dispensable for retro transcytosis of IgA-coated *Salmonella* (16).

However, these studies largely rely on animal studies. The effects of IgA on pathogens have not been fully elucidated in humans. Selective IgA deficiency, which is defined as undetectable levels of IgA in the serum, stool, etc. while other immunoglobulin classes remain intact, is the most common primary immunodeficiency in humans. It affects one in 200 to 1,000 individuals in Caucasians, although the prevalence is varied among populations (9, 10). Although selective IgA deficiency is frequently complicated with other immune-related diseases such as Graves’ disease, type 1 diabetes, and celiac disease (17, 18), it has been reported that IgA deficiency mainly increases the risk of infectious diseases. For example, Jorgensen et al. have reported that IgA deficiency increases the incidence of upper and lower respiratory tract infection and allergic diseases compared with healthy individuals (19). Aytekin et al. have also analyzed 118 patients with selective IgA deficiency in Turkey and revealed that 83.9% of patients develop some infectious diseases during 7 years (median) of the observation period (20). Recurrent infections are observed in up to one-third of symptomatic patients (21). However, these studies also show that critical infectious diseases are not common in patients with selective IgA deficiency, and it appears that its susceptibility to infectious diseases is not as severe as other primary immunodeficiency diseases such as common variable immunodeficiency (22). This fact may be attributable to compensation by other immunoglobulin classes such as IgM. There are five immunoglobulin classes in humans: IgM, IgG, IgA, IgD, and IgE. Although IgA is the most dominant immunoglobulin in the intestine, IgM and IgG are also responsible for regulating intestinal immunity and infections (23, 24). Several reports have revealed that IgM is highly detected in the stool of IgA deficient patients (25), implying the compensatory role of IgM in regulating the intestinal environment. However, the relationship between enhanced IgM secretion and pathogen control in humans has not been fully understood.

Recent studies also highlight the impact of IgA deficiency on gut commensal bacteria (11, 13, 25). As in mice, human IgA can react to gut commensals (26). In addition, Sterlin et al. have specifically addressed the microbial reactivity of two human IgA subclasses: IgA1 and IgA2 (27). Interestingly, both IgA1 and IgA2 similarly bind to small intestinal bacteria, whereas genera from phylum Bacteroidetes, especially *Flavobacterium*, are preferentially bound by IgA2. Potentially concordant with this observation, IgA2 is more common, especially in the colon (28). The major difference between two IgA subclass is the length of hinge region, with IgA1 having an extended hinge, which may confer advantage in antigen binding (29). However, the longer hinge region also allows pathogenic bacteria to cleave and impair IgA1 function through their proteolytic enzymes (30). It has also been reported that IgA1 and IgA2 show different glycosylation profiles, with IgA1 having more sialic acids (31). The difference in the glycosylation profiles seems to affect their functional properties, as serum IgA2 shows more proinflammatory profiles than IgA1. Although these studies did not specifically address the difference of IgA1 and IgA2 in the intestine, we can extrapolate these data to speculate that IgA1 and IgA2 may play a different role in regulating microbiota in humans. To support our speculation, it has been reported that IgA2 but not IgA1 is able to bind to M cells on the Peyer’s patches, which play an important role in initiating the production of antigen-specific IgA (32).

In line with the role of IgA in regulating pathogens, IgA deficiency seems to increase the abundance of pathobionts, i.e. members of commensal bacteria potentially harmful to host homeostasis under certain conditions. For example, Fadlallah et al., have reported that *Escherichia coli*, a known pathobiont and target of IgA binding, are increased in the stool of patients with selective IgA deficiency (11). Similarly, Moll et al. have shown that *E. coli* is one of the major gut colonizers in healthy individuals and more prominent in patients with selective IgA deficiency (13). Although direct associations have not been elucidated, selective IgA deficiency is associated with the elevation of proinflammatory cytokines such as interleukin (IL)-6 and IL-17, which may be attributable to the increase in pathobionts (11). Again, IgM seems to compensate for part of IgA roles in regulating commensal bacteria; however, Enterobacteriaceae including *E. coli* is not targeted by IgM (11). In addition, Magri et al., have shown that dual coating of microbes by SIgA and SIgM is a cardinal feature in humans rather than mice (24), suggesting a compensatory role of IgM. However, the extent of compensation by IgM is not fully understood, as IgM appears to be less specific to gut commensals (25).

Interestingly, many studies have linked IgA deficiency to several non-infectious diseases such as autoimmune diseases (17, 18), which have also been associated with the gut commensal microbiota. It also appears that autoimmunity in turn affects the regulation of microbiota since anti-IgA antibodies in selective IgA deficiency are associated with the expansion of pathobionts like *E. coli* (13). These findings raise the possibility that IgA may modulate the susceptibility of human diseases through the regulation of commensal microbiota, and it encourages researchers to understand the impact of commensal microbiota on our health and how IgA can interact with these bacteria.

## How does IgA regulate the commensal microbes?

We here discuss how IgA interacts with the gut microbiota. Unfortunately, vast evidence in this field is established in mouse experiments. Nevertheless, we think it is possible to extrapolate these experimental findings to human settings, particularly thanks to the recent development of IgA-sequencing (IgA-seq) analysis. Previously, it was necessary to examine the affinity of IgA with microbes one by one, while IgA-seq analysis, which takes advantage of next-generation sequencing techniques, provides an overall picture of IgA bound to commensal microbes and enables researchers to comprehensively understand IgA-microbe interactions in both humans and mice. The detailed molecular mechanisms by which IgA binds to microbial antigens are also discussed elsewhere (7, 8, 33).

In line with the central role of immunoglobulins, IgA mainly regulates the colonization, invasion, growth, and motility of commensal bacteria. The importance of intestinal IgA in the regulation of commensal microbiota is initially recognized by Fagarasan et al. In their study, they used mice deficient in activation-induced cytidine deaminase (AID), a critical regulator of both class switching and somatic hypermutation of the immunoglobulin locus, and thereby lacking immunoglobulins other than IgM. The authors found an overgrowth of gut microbiota in AID deficient mice (34). AID deficiency particularly expands segmented filamentous bacteria (SFB) colonization in the intestine (35). Later, the same group also reported that a point mutation in AID which leads to loss of somatic hypermutation but not class switching resulted in similar consequences (36), suggesting the importance of affinity maturation in immunoglobulins, particularly IgA. Similarly, it has been reported that PD1-deficient mice show decreased IgA-binding bacteria and dysregulation of gut microbiota, which result from abnormal follicular T-cell function (37). More recently, Nagaishi et al. have specifically addressed the alterations of gut microbiota and intestinal homeostasis in IgA-deficient mice (38). They showed a skewed microbiota composition in IgA-deficient mice, including an expansion of SFB. IgA-deficient mice also exhibited spontaneous inflammation in the ileum, which was canceled by antibiotic treatment. These studies indicate that IgA, especially those produced by the T cell-dependent pathway and therefore characterized by its high affinity, could functionally regulate the growth of intestinal bacteria, especially pathobionts.

With the widespread use of IgA-seq techniques, it has become recognized that the patterns of IgA-binding bacteria in specific diseases and environments are different. Palm et al. first showed in 2014 that the pattern of IgA-binding bacteria is altered in patients with IBD and that colonization of IgA-binding bacteria from IBD patients but not healthy individuals in germ-free mice exacerbates intestinal inflammation in the colitis model (39). Similarly, IgA binding to pathogenic *E. coli* is increased in the feces of patients with spondyloarthritis (40). IgA-coated bacteria are also altered in multiple sclerosis (41, 42), kwashiorkor (43), obesity after bariatric surgery (44), and certain types of cancer (45). These results suggest that IgA may have the ability to bind to and control potentially harmful commensal bacteria that are involved in human pathogenesis.

It is also important to note that IgA can activate the immune system against gut microbes *via* retro transcytosis into the Peyer’s patches (15). Although antigens derived from pathogens are transported through M cells scattered in the epithelium covering the Peyer’s patches and recognized by dendritic cells located right beneath M cells, several studies show that M cell-dependent transcytosis also appears important for commensal bacteria such as *E. coli* and SFB (46–48). Mikulic et al. have experimentally shown that IgA coating of *Lactobacillus rhamnosus*, a symbiotic gut microbe, plays a role in conditioning dendritic cells, which facilitates tolerogenic responses such as induction of regulatory T cells (49). These findings suggest that IgA not only regulates commensal bacteria through a direct interaction but also affects the gut environment by modulating the intestinal immune system through M cell-mediated uptake of commensal microbes.

Interestingly, IgA can promote the colonization of gut microbes in certain situations. This is especially true for *Bacteroides*, a major symbiotic genus in the gut. For example, Nakajima et al. have reported that IgA facilitates colonization of *B. thetaiotaomicron* (50). As a mechanism, the authors have suggested that IgA alters the gene expression of *B. thetaiotaomicron* related to polysaccharide utilization required for colonization in the mucus layer, which they named Mucus-Associated Functional Factor (MAFF). Another report has shown that IgA fosters stable colonization of *B. thetaiotaomicron* by altering its epitope expressions and thereby silencing excessive inflammatory responses (51). Donaldson et al. have reported that IgA mediates the adhesion of *B. fragilis* to intestinal epithelial cells in a capsular polysaccharide-dependent manner (52). IgA is also found to facilitate biofilm formation of *E. coli* in an *in vitro* setting (53, 54). The mechanism by which IgA regulates commensal microbes in opposite ways is not fully understood; however, it has been proposed that adhesive molecules produced by the host (e.g., IgA and mucus) and mucus flow could select for and against certain microbes (55). In addition, replication rates of microbes and the bound break of IgA enchainment may also explain this difference (56, 57). In this model, the host produces IgA to all types of bacteria; however, those characterized by higher replication rates may remain enchained even after their division, since their replication rates are faster than SIgA-crosslinking breaks of enchained clusters. This model may explain why SIgA is likely to affect fast-growing bacteria such as pathobionts and eliminate them from the intestine.

It is generally recognized that the synthetic pathways of IgA largely determine its functional characteristics. The T cell-dependent pathway is supported by CD4 T cells that results in differentiation of high-affinity IgA-producing cells, while the T cell-independent pathway is mainly supported by dendritic cells and results in the production of low-affinity IgA (58–60). There is evidence showing that the high affinity IgA resulting from T-cell help and somatic hypermutation is the cardinal feature of IgA function in regulating gut microbiota. For example, as we discussed earlier, a point mutation in AID that impairs somatic hypermutation elicits dysregulation of gut microbiota (36). Kabbert et al. have also analyzed the characteristics of human IgA and reported that somatic hypermutation rather than polyreactivity, another aspect of IgA, is associated with microbial reactivity (26). Furthermore, Okai et al. have reported that oral administration of high-affinity IgA can alter gut microbiota composition (61). In a report by Bunker et al., the authors compared the microbiota composition in wild-type and T cell-deficient mice and showed that IgA produced through the T cell-dependent pathway was capable of binding to microbes that colonize close to epithelial cells, such as SFB and *Mucispirillum* (62). It has been proposed that although these bacteria are generally considered as pathobionts, their proximity to epithelial cells makes them more likely to be captured by antigen-presenting cells and consequently more likely to be presented their antigens to CD4 T cells, leading to the production of high-affinity IgA toward them in the T cell-dependent pathway (62). Indeed, mice with impaired T-cell function, including those deficient in T-cell receptor beta and delta chains, have altered affinity and specificity of IgA, resulting in changes in the composition of intestinal bacteria (37, 62, 63). Yang et al. have also reported that IgA specificity to commensal microbe is dependent on CD4 T cells (63). We similarly reported that acetate, one of the SCFAs, is potent in enhancing T cell-dependent IgA production and that it has a substantial impact on the composition of mucosa-associated bacteria in an IgA-dependent manner (64). We also showed that acetate facilitates the clearance of *E. coli* in the mucus layer, which also appears dependent on its IgA coating. On the other hand, the role of IgA produced by the T cell-independent pathway in regulating gut microbiota has not been well elucidated. It is widely considered that T cell-independent IgA generally binds to a wide variety of bacterial antigens.

Meanwhile, an IgA repertoire analysis reveals that V gene usage among IgA-producing B cells in the intestine is restricted and that somatic hypermutation profile is less likely shared among clonally related cells, suggesting that antigen selection and affinity maturation may be uncommon (65). In addition, IgA-coated bacteria are detected in T-cell deficient mice to a similar extent to their wild-type counterpart despite a severe reduction in SIgA (60, 62, 66), suggesting a capability of T cell-independent IgA in binding commensal bacteria. Polyclonal and low affinity antibodies, mainly IgE and IgG1, are also considered to play a role in host defense against certain gut pathogens such as helminth (67). These studies may support the role of low-affinity IgA in regulating gut homeostasis. Nevertheless, the role of T cell-independent IgA in regulating gut bacteria has not been fully elucidated. It has been recognized that T cell substantially impacts microbiota composition through IgA production (68). Furthermore, Grasset et al. showed that IgA in TACI-deficient mice, which lack T cell-independent IgA, did not alter gut microbiota in the colonic tissue and stool contents (60). These studies show that although T cell-independent IgA can bind to gut microbes, its role in regulating microbiota composition, in contrast to T cell-dependent IgA, remains elusive. Although not proven, this may be attributable to the low affinity of T cell-independent IgA, since sufficient affinity to gut bacteria appears required for their discharge from the intestinal lumen (56, 57). It is also important to note that apart from somatic hypermutation, N-linked glycosylation of the variable domain in antibody also affects antigen binding (69). In addition, the affinity of glycan-glycan interactions with bacterial components such as lipopolysaccharides could be sufficiently high (70). Although this is a hypothesis requiring future investigation, glycan-glycan interactions may be another perspective of IgA in regulating microbiota. Taken together, the function of IgA on the gut microbiota is likely to be defined by its mode of induction and structure, and in particular, its affinity appears to have a significant impact.

**Figure 1:**
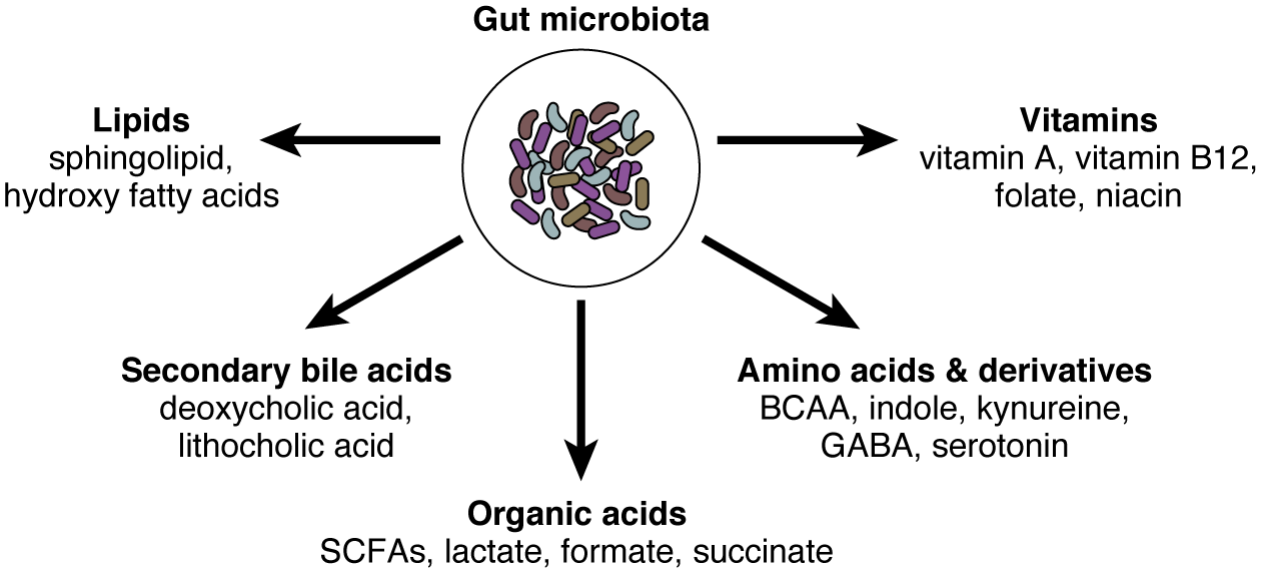
The gut microbiota and their small molecules. The gut microbiota possesses millions of genes and produces thousands of small molecules. Here we summarized representative microbe-derived small molecules, including organic acids, amino acids and their derivatives, vitamins, secondary bile acids, and lipids. SCFAs, short-chain fatty acids; BCAA, branched-chain amino acids; GABA, gamma-aminobutyric acid.

Another important aspect is the regulation of IgA-microbe interactions by environmental factors. Microbe-derived small molecules such as metabolites are important regulator of intestinal immunity (**Figure 1**). As we discussed above, microbial metabolites (e.g., SCFA) augment intestinal IgA production and regulate their reactivity with microbes (64, 71, 72). Other dietary components and nutrients such as dietary antigens (73), glutamine (74), vitamin A (75, 76), and dietary fat (77) are also considered important regulators of intestinal IgA. ATP, which could be released during tissue damage and presumably during the invasion of pathogens, is another important environmental stimulus that regulates IgA production and mucosal colonization (78). Nutrient status can also impact gut microbiota and their properties of being bound by IgA. For example, under starvation, *Lactobacillus* spp. that minimally express surface antigens for IgA binding can selectively expand, resulting in altered IgA-microbe interrelationships at the community level (79).

Finally, recent studies also appreciate the commensal fungi as important modulators of the immune system and host diseases such as IBD (80–82). Importantly, IgA appears to regulate the commensal fungi as well. For example, Ost et al. have shown that intestinal IgA can bind to and suppress hyphae of *Candida albicans*, which can be potentially harmful to intestinal homeostasis (83). Interestingly, IgA reactive with *C. albicans* induced by a vaccination can reduce the severity of DSS-induced colitis. Similarly, Doron et al. have also reported that SIgA in mice and humans preferentially binds to *C. albicans* hyphae (84). Notably, SIgA reactive with hyphae-producing antigens such as Sap6 and candidalysin were significantly reduced in the mucosa of Crohn’s disease, implying a protective role of candida-specific IgA in disease pathogenesis.

## Potential mechanisms by which intestinal IgA regulates host pathophysiology

Considering that gut microbiota has tremendous impacts on human physiology and pathology and that IgA effectively alters the composition of the microbiota, it is tempting to assume that IgA may modulate the pathogenesis of intestinal and extra-intestinal diseases in humans. Understanding the underlying mechanisms by which IgA impacts disease susceptibility may also be important for the clinical application of IgA in future. As most human findings in IgA-microbe interactions are purely associative and correlative, we discuss experimental findings to extrapolate the role of IgA in regulating microbiota-mediated diseases.

### Intestinal diseases

As we discussed above, it is not surprising that intestinal diseases such as IBD are more prominently influenced by the gut microbiota. It has been shown that IBD is characterized by overgrowth of pathobionts such as *E. coli* and *Klebsiella* in the intestine, which may be functionally involved in the progression of disease activity and complications (39, 40, 85–87). These bacteria seem to directly penetrate the mucosal layer and thereby excessively fuel immune reactions (39). In line with the protective role of IgA against pathobionts, many human studies have revealed that IgA is bound preferentially to pathobionts and/or colitogenic bacteria in IBD (39, 40, 88). A report has also shown that SIgA is increased in IBD (89), implying that IgA is produced in response to the expansion of pathobionts. Furthermore, a recent paper by Shapiro et al., revealed that IgA coating to *Oscillospira* is linked with a delay in time to surgery in IBD patients, suggesting that IgA-coated bacteria can be clinically applied as an IBD biomarker (88). Together, these human findings warrant the application of IgA to IBD for therapeutic and/or diagnostic purposes. In this regard, Okai et al. have reported that administration of a high affinity and polyreactive IgA, named W27, can bind to a wide variety of gut commensal bacteria including pathobionts and that this high-affinity IgA can mitigate colitis in experimental models (61). Similarly, Xiong et al. have shown that oral administration of W27 ameliorates a colitis model in marginal zone B and B-1 cell-specific protein (MZB1)-deficient mice, which largely impairs the secretion of IgA into the gut lumen (90). The effects of W27 have also been tested in an *in vitro* model of the human microbiota, with a prominent effect on the growth inhibition of *E. coli* (91). These findings suggest that limit of bacterial growth and/or faster discharge from the intestinal lumen is facilitated by exogenous IgA administration, and that this may serve as a protection against colitis. However, it is important to note that IgA binding to pathobionts is not always a good thing; for example, in Crohn’s disease, NOD2 mutation facilitates retro transcytosis of IgA-coated bacteria through M cells, which may fuel intestinal inflammation and permeability (92).

As we discussed above, commensalism of other microorganisms such as fungi may also regulate the disease severity and outcome of IBD (83, 84). Virus is yet another pathogen that is controlled by IgA. The regulation of virus by SIgA is particularly important for protection against enteropathogenic virus such as norovirus and rotavirus (93–95). Rotavirus vaccination failure is associated with lower plasma rotavirus-specific IgA (96), suggesting that IgA induction through vaccination functionally protect against rotavirus infection. How IgA regulates commensal virome has not been fully elucidated. Although many studies have suggested altered gut viromes in IBD patients (97–99), one study show that IgA deficiency does not substantially impact viral profile at least in the saliva (100).

In addition to IBD, it has been recognized that irritable bowel syndrome (IBS) is also associated with altered gut microbiota and their metabolites (101, 102), which is characterized by an increase in pathobionts such as the family Enterobacteriaceae (101). Both SIgA and IgA-coated bacteria are increased in IBS patients, especially those of diarrhea-predominant type (IBS-D) (103, 104). It also appears that IgA coating toward the genus *Escherichia*–*Shigella* is particularly promoted in patients with IBS-D and is positively correlated with some clinical manifestations of IBS such as anxiety and depression scores (103). These findings suggest that, like IBD, IgA may be produced and secreted upon an expansion of pathobionts in IBS, although a protective role of IgA in the pathogenesis of IBS remains elusive.

### Cardiometabolic diseases

Cardiometabolic diseases is another good example of how the regulation of microbiome by IgA can be a potential treatment strategy. For example, IgA deficiency in a mouse model of obesity (e.g., high-fat diet-induced obesity) aggravates the metabolic phenotypes (77). In addition, IgA positively correlated with the improvement of metabolic parameters in the patients who underwent bariatric surgery (77). Interestingly, serum IgA has shown to increase in patients with type 2 diabetes, although fecal IgA has not been studied (105, 106). These human studies raise the possibility that IgA may somehow affect the pathogenesis of cardiometabolic diseases. As we noted above, translocation of microbial components such as LPS and flagellin elicits low-grade inflammation in the liver and adipose tissue, resulting in insulin resistance and obesity (107, 108). In this regard, it has been reported that vaccination with flagellin is potent in regulating the localization of gut microbes through induction of flagellin-specific IgA (109). Furthermore, the vaccination ameliorates colitis and diet-induced obesity. Fujimoto et al. have similarly reported that immunization with a microbial antigen with adjuvants (i.e. CpG and curdlan) shows potent induction of anti-*Clostridium ramosum* IgA in the intestine, and this specific antibody can ameliorate obesity and other metabolic consequences (110). Since microbe-derived metabolites also play an important role in the development of cardiometabolic diseases, the regulation of microbial transcriptional activity could be another therapeutic strategy. In this regard, several papers reveal that host immune reactions including IgA binding to gut microbes also alter the transcriptional activity and metabolite production by gut microbes (50, 111). As we discussed earlier, T-cell help is important for the production of high-affinity IgA that can regulate microbiota. In this regard, Petersen et al. have revealed that MyD88 deficiency in T cells, which results in impaired T-cell help function in producing intestinal IgA, aggravates obesity in a microbiota-dependent manner (112). Collectively, these findings suggest that IgA is involved in the pathogenesis of metabolic diseases and that IgA can be a useful toolbox to alter the gut microbiota and thereby host metabolism.

It should be stressed, however, that metabolic phenotypes have not been reported in patients with IgA deficiency. We therefore consider that IgA deficiency itself may minimally contribute to host metabolism in humans, possibly due to compensatory mechanisms by other immunoglobulin classes and/or other factors influencing host metabolism (e.g., recurrent infections). Nevertheless, this does not exclude any possibilities that an IgA-based intervention targeting certain commensal bacteria could effectively alter the microbial community and thereby host metabolic diseases. Needless to mention, more studies are required to prove the concept in future.

### Liver diseases

Gut microbes can penetrate and translocate into the host body in certain conditions such as obesity and diabetes (113–115). Therefore, the role of IgA as a defense against microbes seems also important in organs other than the intestine. The liver is considered a firewall against bacterial translocation. Moro-Sibilot et al. have revealed that IgA-producing plasma cells are distributed in the human liver, and liver-derived IgA appears to be highly reactive to *B. vulgatus*, a human commensal species (116). They also show in mice that these IgA-producing cells in the liver are derived from the Peyer’s patches. However, in an alcohol-induced hepatitis model, they report that an increase in liver IgA-producing cells is associated with worse outcomes and that suppressing the migration of IgA-producing cells from the Peyer’s patches to the liver can ameliorate liver injury. Therefore, although IgA produced in the liver seems to react with the commensal bacteria, the functional role in terms of IgA-bacteria interactions, especially in the liver, remains elusive.

### Inflammation in the central nervous system

IgA is reportedly involved in autoimmune diseases such as multiple sclerosis (MS). However, unlike IBD or cardiometabolic diseases, it seems that IgA-producing cells themselves may affect the pathogenesis of experimental autoimmune encephalomyelitis (EAE), a disease model for MS, by producing immune suppressive cytokines such as IL-10 (117). Interestingly, the authors also showed a reduction of IgA-coated bacteria in severe MS patients, which is also confirmed in another report (41). In addition, they experimentally proved that increase in intestinal and brain IgA by the colonization of *Trichomonas musculis*, an intestinal protozoan, ameliorated EAE susceptibility independently of T-cell subsets such as Th1 and Th17 (117). Although this paper did not address the IgA reactivity with specific bacteria, another study characterized the bacterial reactivity of IgA in the central nervous system in MS patients (42). In this study, the authors showed that IgA-producing cells were reactive with a diverse array of gut microbiota but not with self-antigens including those of human brains. These findings reveal an intimate interaction between the gut and central nervous system in MS, although the migratory mechanisms have not yet been elucidated. It also remains elusive whether the specificity of IgA to certain commensals affects the disease susceptibility.

### Aging

Aging is characterized by low-grade inflammatory responses in multiple organs. Since it is also associated with sequential alterations of gut commensal microbiome, many researchers consider that microbes may be a source of inflammation during the aging process. Interestingly, not only the commensals but also IgA-coated bacteria are altered during aging. Sugahara et al. have reported that while Bifidobacteriaceae was reduced in the adult group (35 years old on average), Enterobacteriaceae and Clostridiaceae were increased in the aged group (76 years old on average) (118), implying that consistent with other reports (119), dysbiosis is progressively aggravated during aging. Notably, IgA binding to Enterobacteriaceae and Clostridiaceae was significantly reduced in the aged group despite that the total IgA amount in the stool remained unchanged (118). This may explain why these pathobionts flourished in the aged group. It has been reported that antigen uptake and antigen-specific immune responses in the intestine are impaired with aging (120–123). Although immunosenescence, i.e. age-related impairment of immune cell functions, is generally considered intrinsic to host organs, tissues and cells, diminished antigen-specific responses may also be attributable to the alterations in the gut environment as “beneficial” signals for immune homeostasis from the intestinal lumen such as SCFAs is reduced with aging (124). More studies are required to unveil the interaction between gut microbiome and immunosenescence and how this could impact the host pathophysiology.

## Potential of IgA-oriented strategies in human diseases

**Figure 2:**
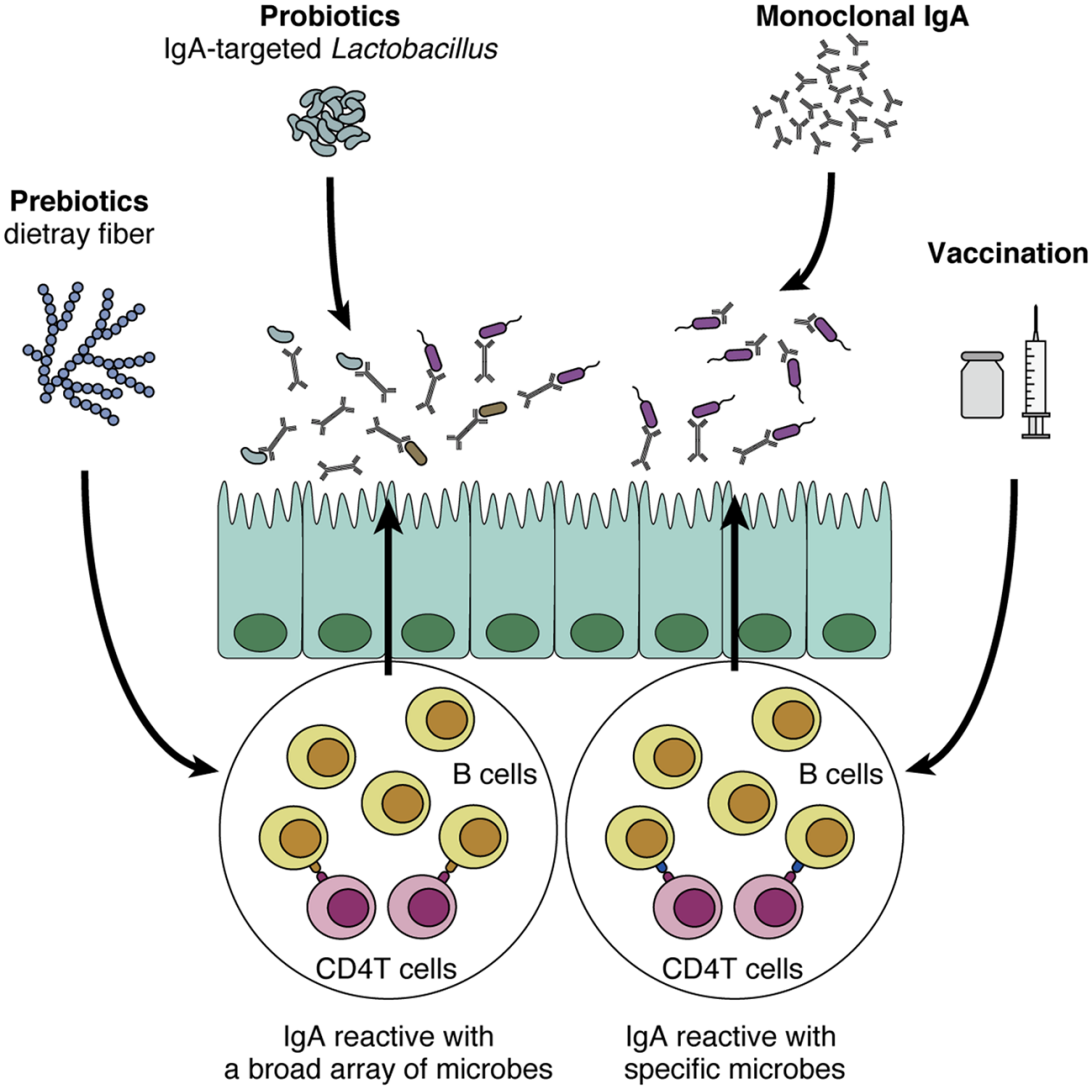
Potential application of IgA to human diseases. IgA-oriented therapies that modulate the gut microbiota could be utilized to control disease susceptibility. First, prebiotics (e.g., dietary fiber) and probiotics increase intestinal IgA production and modulate IgA-coating bacteria. In turn, IgA-coated *Lactobacillus* could be isolated and harnessed as a probiotic strain that directly interacts with the mucus layer and epithelial cells. Although these methods are relatively easy to implement, their effects on the intestinal immune system are not specific to IgA production. In addition, it is difficult to induce IgA reactive with specific microbes of interest. By contrast, vaccination and monoclonal IgA administration are more sophisticated in the sense that these methods leverage “specificity” toward certain microbes, a cardinal feature of adaptive immunity. As the efficacy of these methods has been shown in mouse models (e.g., colitis and high-fat diet-induced obesity), these approaches that target specific microbes of interest would be promising.

Given the promising roles of IgA in regulating and modulating the gut commensal microbiota, it is tempting to assume that IgA can be a novel therapeutic tool for many human diseases. Here we propose four directions for future development of IgA-oriented therapies (**Figure 2**). First, prebiotics and probiotics can be harnessed to augment intestinal IgA, although it has various effects on the mucosal immune system (125, 126). As we discussed earlier, prebiotics such as dietary fibers that increase intestinal SCFA levels can potently augment intestinal IgA (71), in particular acetate consistently shows this effect as reported in several papers (64, 72). In addition, other prebiotics including fructo- and galacto-oligosaccharides also show robust effects on mucosal IgA response in mice and humans (127–129). This seems relatively easy for application since all we need is to consume these fibers and/or SCFAs. However, a problem would be that prebiotics cannot specify the target microbes of IgA. In addition, many papers show that the effect of dietary fiber is highly variable, probably due to the differences in individual microbiota profiles (130, 131), suggesting that it might be difficult to predict IgA responses after administration of the dietary fibers.

The effect of probiotics on IgA production and secretion has also been extensively researched. Lactic acid bacteria such as *Lactobacillus* and *Pediococcus* strains have been shown to increase IgA production by augmenting dendritic cells to enhance secretion of IL-6 and/or IL-10, important cytokines for IgA production (132, 133). Another study reveals that oral administration of a heat-killed *Lactobacillus* strain increases antigen-specific IgA secretion in OVA-immunized mice, presumably through a T cell-dependent manner (134). In addition, oral administration of *Lactobacillus* and *Bifidobacteria* strains to formula-fed infants increases their cow milk-specific IgA-producing cells in blood samples (135). These findings suggest that application of probiotic strains may augment IgA secretion and function to protect barrier function. From a different perspective, considering that IgA-seq analysis can distinguish a certain group of microbes, we may be able to isolate an IgA-coated microbe as a next-generation probiotic strain that possesses interesting functions in the intestine. In this regard, Sun et al. have analyzed the differences between IgA-coated and -uncoated *Lactobacillus* and shown that IgA-coated species can modulate gut barrier function (136).

The adaptive immune system, and more specifically T-cell help, is recognized as a cardinal component for specific IgA production, which is important for the regulation of pathobionts in the intestine. From this perspective, triggering the adaptive immune system with vaccination *via* either oral or systemic route is another possible strategy for IgA-oriented therapy. For example, immunization of flagellin *via* intraperitoneal injection is efficacious in inducing anti-flagellin IgA in mice (109). Interestingly, this systemic immunization also alters gut microbiota and several microbiota-associated host pathologies such as high-fat diet-induced obesity (109). In addition, Fujimoto et al. have shown that intramuscular injection of antigens with adjuvants could efficiently induce antigen-specific IgA responses (110). They showed that vaccination with *C. ramosum*-derived antigens induced anti-*C. ramosum-*specific IgA in the feces and reduced the abundance of this microbe in the colonic mucosa, while this vaccination did not alter the microbial community structure. Given that mucosal vaccination is believed to efficiently induce IgA especially and effectively protect against respiratory diseases such as influenza virus infection (137), the development of a mucosal vaccination strategy that regulates the microbiota may also be useful.

Monoclonal antibodies targeting specific molecules have been clinically applied to many human diseases such as cancers for many years (138). Likewise, monoclonal IgA antibodies that show potent effects on the gut microbiota could be selectively expanded and administered orally to the patients. This concept was first shown by Okai et al.; as we discussed above, they nicely revealed the efficacy of high-affinity and polyreactive IgA in regulating the gut microbiota and improving colitis after its oral administration (61). Another group has also shown a similar effect of oral monoclonal IgA administration (90). In a *Salmonella* infection model, oral administration of monoclonal IgA named Sal4 efficiently blocks the invasion of *Salmonella* into the Peyer’s patches (139). As both vaccination and oral administration are sophisticated strategies leveraging cardinal features of adaptive immunity, i.e. high affinity and specificity especially with pathobionts, it is tempting to anticipate future application to human diseases relating to gut microbiota. For further development, it also seems important to address the safety and stable delivery of vaccines/IgA in humans and to define target antigens/microbes appropriate for vaccines/IgA. Taken together, these pioneering studies point to a promising possibility that IgA can be exploited as an efficient regulator of the gut commensal microbiota.

## Conclusions

In this review, we discuss how IgA regulates microbiota in the intestine and how this interrelationship impacts host physiology and pathology, especially in the context of human homeostasis. IgA-based strategies aiming to modulate the gut microbiota such as prebiotics, vaccination, and exogenous administration have been increasingly studied and regarded as a promising therapeutic toolbox for human diseases mediated by the gut microbiota. Nevertheless, much evidence of IgA-microbe interactions has been established based upon experimental findings, emphasizing the necessity to explore the role of IgA in humans. In this regard, a clinical application of IgA-based strategies may serve not only therapeutic purposes but also human evidence to show causality between IgA and the gut microbiota in basic science, which would further accelerate our understanding of human pathophysiology at the system level.

## Author contributions

TT and HO conceptualized the work and co-wrote the manuscript. All authors contributed to the article and approved the submitted version.

## Funding

This work is supported by internal research funds from RIKEN.

## Acknowledgments

We thank Drs. Takashi Kanaya, Eiji Miyauchi, and Yumiko Nakanishi for their helpful comments.

## Conflict of interest

The authors declare that the research was conducted in the absence of any commercial or financial relationships that could be construed as a potential conflict of interest.

## Publisher’s note

All claims expressed in this article are solely those of the authors and do not necessarily represent those of their affiliated organizations, or those of the publisher, the editors and the reviewers. Any product that may be evaluated in this article, or claim that may be made by its manufacturer, is not guaranteed or endorsed by the publisher.
